# Generation of NKX2.5^GFP^ Reporter Human iPSCs and Differentiation Into Functional Cardiac Fibroblasts

**DOI:** 10.3389/fcell.2021.797927

**Published:** 2022-01-21

**Authors:** Leyre López-Muneta, Javier Linares, Oscar Casis, Laura Martínez-Ibáñez, Arantxa González Miqueo, Jaione Bezunartea, Ana Maria Sanchez de la Nava, Mónica Gallego, María Eugenia Fernández-Santos, Juan Roberto Rodriguez-Madoz, Xabier L. Aranguren, Francisco Fernández-Avilés, José Carlos Segovia, Felipe Prósper, Xonia Carvajal-Vergara

**Affiliations:** ^1^ Regenerative Medicine Program, Foundation for Applied Medical Research (CIMA), Instituto de Investigación Sanitaria de Navarra (IdiSNA), University of Navarra, Pamplona, Spain; ^2^ Departament of Physiology, Faculty of Pharmacy, University of the Basque Country UPV/EHU, Vitoria-Gasteiz, Spain; ^3^ Program of Cardiovascular Diseases, Foundation for Applied Medical Research (CIMA), Instituto de Investigación Sanitaria de Navarra (IdiSNA), University of Navarra, Pamplona, Spain; ^4^ Centro de Investigación Biomédica en Red Cardiovascular (CIBERCV), Instituto de Salud Carlos III, Madrid, Spain; ^5^ Retinal Pathologies and New Therapies Group, Experimental Ophthalmology Laboratory, Department of Ophthalmology, University of Navarra Clinic, Pamplona, Spain; ^6^ Department of Cardiology, Hospital General Universitario Gregorio Marañón, Instituto de Investigación Sanitaria Gregorio Marañón (IISGM), Madrid, Spain; ^7^ Centro de Investigación Biomedica en Red de Enfermedades Cardiovasculares (CIBERCV), Madrid, Spain; ^8^ Hemato-oncology Program, CIMA Universidad de Navarra, Instituto de Investigación Sanitaria de Navarra (IdiSNA), Centro de Investigación Biomédica en Red de Cáncer (CIBERONC), Pamplona, Spain; ^9^ Facultad de Medicina, Universidad Complutense de Madrid, Madrid, Spain; ^10^ Cell Technology Division, Centro de Investigaciones Energéticas, Medioambientales y Tecnológicas (CIEMAT), Centro de Investigación Biomédica en Red de Enfermedades Raras (CIBERER), Madrid, Spain; ^11^ Unidad Mixta de Terapias Avanzadas, Instituto de Investigación Sanitaria Fundación Jiménez Díaz (IIS-FJD, UAM), Madrid, Spain; ^12^ Centro de Investigación Biomédica en Red de Cáncer (CIBERONC), Instituto de Investigación Sanitaria de Navarra (IdiSNA), Department of Hematology and Cell Therapy, University of Navarra Clinic, Pamplona, Spain

**Keywords:** induced pluripotent stem cells, reporter, cardiac, fibroblasts, direct reprogramming

## Abstract

Direct cardiac reprogramming has emerged as an interesting approach for the treatment and regeneration of damaged hearts through the direct conversion of fibroblasts into cardiomyocytes or cardiovascular progenitors. However, in studies with human cells, the lack of reporter fibroblasts has hindered the screening of factors and consequently, the development of robust direct cardiac reprogramming protocols.In this study, we have generated functional human NKX2.5^GFP^ reporter cardiac fibroblasts. We first established a new NKX2.5^GFP^ reporter human induced pluripotent stem cell (hiPSC) line using a CRISPR-Cas9-based knock-in approach in order to preserve function which could alter the biology of the cells. The reporter was found to faithfully track NKX2.5 expressing cells in differentiated NKX2.5^GFP^ hiPSC and the potential of NKX2.5-GFP + cells to give rise to the expected cardiac lineages, including functional ventricular- and atrial-like cardiomyocytes, was demonstrated. Then NKX2.5^GFP^ cardiac fibroblasts were obtained through directed differentiation, and these showed typical fibroblast-like morphology, a specific marker expression profile and, more importantly, functionality similar to patient-derived cardiac fibroblasts. The advantage of using this approach is that it offers an unlimited supply of cellular models for research in cardiac reprogramming, and since NKX2.5 is expressed not only in cardiomyocytes but also in cardiovascular precursors, the detection of both induced cell types would be possible. These reporter lines will be useful tools for human direct cardiac reprogramming research and progress in this field.

## Introduction

Cardiovascular diseases, including those associated with myocardial infarction (MI), remain the leading cause of morbidity and mortality worldwide, causing more than 30 and 40% of total deaths in the United States and European countries, respectively. (Timmis et al., 2020; Virani et al., 2021). MI produces the irreversible loss of a large part of the working myocardium that over the last 2 decades investigators have tried to replace with adult stem cells and stem cell-derived cardiac and vascular cell transplantation. (Steinhauser and Lee, 2011; Cambria et al., 2017).

Human pluripotent stem cells, including human embryonic stem cells and induced pluripotent stem cells (hiPSC), offer the opportunity to obtain any cell type in the body, including cardiac cells, and are actively being investigated as a source of cardiac cell replacement. However, the risk of tumorigenesis and the time-consuming nature of the procedure required to establish hiPSC from patients and to obtain differentiated lineages limit their therapeutic application. The direct cell reprogramming of cardiac fibroblasts (cFib), that ultimately lead to the formation of a scar after myocardial infarction, (Steinhauser and Lee, 2011), into cardiac lineages could circumvent these issues.

Direct conversion of mouse fibroblasts, extracted from *αMHC*-GFP reporter mice, into induced cardiomyocytes (iCM) was first achieved *in vitro* by overexpression of the GATA4, MEF2C and TBX5 transcription factors (GMT cocktail). (Ieda et al., 2010). However, this cocktail was demonstrated to be unable to promote reprogramming of human fibroblasts, and the inclusion of two or more additional factors was necessary. Further, unlike mouse iCM, in most studies human iCM did not show spontaneous beating (Fu et al., 2013; Nam et al., 2013; Wada et al., 2013; Muraoka et al., 2014; Christoforou et al., 2017). The requirement of additional factors diminishes the transduction efficiency and limits *in vivo* delivery in one single vector. This is important to take into consideration since direct cardiac reprogramming *in vivo* is considered a more feasible therapeutic approach than *in vitro*, as it has shown a higher reprogramming efficiency (Qian et al., 2012; Song et al., 2012) and can circumvent one of the major problems of cell therapy, namely poor engraftment. (López-Muneta et al., 2020). Alternatively, enhancement of reprogramming efficiency to obtain human iCM would be critical to produce sufficient cells *in vitro* for transplantation. Interestingly, expandable multipotent cardiovascular progenitors, analogous to embryonic cardiogenic mesoderm progenitors with the potential to differentiate not only into CM but also into vascular lineages, were obtained using fibroblasts derived from a *Nkx2.5*-EYFP reporter mouse by direct reprogramming, (Lalit et al., 2016), but this has not yet been achieved in human cells. In any case, a great deal more investigation is required before direct cardiac reprogramming approaches can be translated into the clinic.

In the studies in which direct conversion was successfully achieved in mouse cells, fibroblasts derived from cardiac reporter mice were used and therefore reprogrammed cells could be easily monitored by the fluorescent protein expression that was under control of a specific endogenous cardiac promoter. (Ieda et al., 2010; Song et al., 2012; Lalit et al., 2016). However, in most human studies, fibroblasts did not carry a bona fide reporter system (Nam et al., 2013; Wada et al., 2013; Muraoka et al., 2014; Christoforou et al., 2017) to detect reprogrammed cells which makes the screening of factors extremely difficult. This might be one of the major reasons why it has not yet been possible to establish robust and reproducible direct cardiac reprogramming protocols in human cells.

The homeobox-containing transcription factor *Nkx2.5*, becomes expressed upon cardiac crescent formation in the early first heart field and second heart field in cardiac development, (Lyons et al., 1995; Zhang et al., 2014), and it is present in late multipotent cardiovascular progenitors and CM. (Elliott et al., 2011; Den Hartogh et al., 2015).

In this study, we have established human NKX2.5^GFP^ cardiac fibroblasts (cFib). We generated a new NKX2.5^GFP^ reporter hiPSC line using a CRISPR-Cas9 system without disrupting the reading frame. These NKX2.5^GFP^ hiPSC were completely characterized and the reporter reliability for tracking NKX2.5 expressing cells was demonstrated. Then NKX2.5^GFP^ cFib were obtained through directed differentiation. Human NKX2.5^GFP^ cFib showed typical fibroblast-like morphology, a specific marker expression profile and functionality analogous to patient-derived cFib.

## Methods

### Cell Culture Maintenance

The hiPSC line CBiPS1sv-4F-5 (abbreviated here as CBiPS5) was established from CD133^+^ cells derived from human cord blood by our group. (Arellano-Viera et al., 2019). Feeder free hiPSC were maintained in mTESR medium (STEMCELL Technologies) supplemented with 100 U/mL Penicillin, 100 μg/ml Streptomycin (Pen-Strep, from Gibco) on 6-well plates precoated with Matrigel GFR (Corning), diluted 1:100 in cold KnockOut DMEM (Gibco) for 1 h (h) at room temperature (RT). Confluent hiPSC were passaged as single cells by enzymatic disaggregation using TrypLE Express reagent (Gibco) at 37°C for 5 min (min) and plated onto new Matrigel coated 6-well plates at a 1:6 ratio in mTesR supplemented with 5 µM ROCK inhibitor (Y-27632 dihydrochloride, STEMCELL Technologies) for 24 h after replating. The medium was changed every other day.

HFF-1 (SCRC-1041™) and BJ (CRL-2522™) foreskin fibroblast cell lines were procured from American Type Culture Collection (ATCC). HDF-a dermal fibroblast cell line was purchased from Sigma-Aldrich. All these cell lines were maintained on EmbryoMax ultrapure water 0.1% gelatin (Millipore) coated dishes and cultured in fibroblast medium [DMEM high glucose (Sigma-Aldrich) supplemented with 10% FBS, 2 mM Glutamax, 0.1 mM NEAA and Pen-Strep, all from Gibco]. The medium was changed every 3 days. When human fibroblasts reached about 90% confluence, cells were harvested using TrypLE Express reagent (Gibco) for 10 min at 37°C. The split ratio used to passage cells was from 1:2 to 1:5. Primary human cFib from discarded surgical tissue from patients undergoing surgery were obtained from the University of Navarra Clinic. This protocol (ref. 096/2012) was approved by Research Ethics Committee of the University of Navarra. Patient cFib were maintained in fibroblast medium supplemented with 10 ng/ml bFGF (Peprotech) up to passage five and the medium was changed every 3 days. When human fibroblasts reached about 90% confluence, cells were passaged at 1:3 ratio using 0.025% Trypsin 0.01% EDTA Solution (Gibco) for 7 min at 37°C.

All cell cultures were maintained at 37°C in a humidified incubator with 5% CO_2_ and 20% O_2_.

### Transduction of BJ Fibroblasts With Human TERT

For the production of lentivirus containing TERT, five million 293 T cells (CRL-3216™) from ATCC were plated on a 15-cm plate 2 days before transfection. Cells were transfected with a 9 µg pLOX-TERT-iresTK vector (Salmon et al., 2000) together with 6 µg psPAX2 and 3 µg pMD2. G lentiviral packaging and envelope plasmids, respectively, using Lipoctamine 2000 Transfection Reagent (ThermoFisher scientific) following the manufacturer´s indications. All three plasmids were gifts from Didier Trono. pLOX-TERT-iresTK (Addgene plasmid #12245; http://n2t.net/addgene:12245; RRID:Addgene_12,245); psPAX2 (Addgene plasmid #12260; http://n2t.net/addgene:12,260; RRID:Addgene_12260); pMD2. G (Addgene plasmid # 12,259; http://n2t.net/addgene:12259; RRID:Addgene_12259).

Virus containing supernatant was collected 3 days after transfection, centrifuged at 2,000 g for 10 min and filtered through a 0.45 µm PVDF Membrane Filter Unit (Millipore). Viral particles were concentrated using Amicon Ultra Centrifugal Filter Units (Millipore). BJ fibroblasts were plated on a 6-well plate 1 day prior to infection at a 75,000 cells/well density. BJ fibroblasts were infected with 100 µL of concentrated lentivirus containing pLOX-TERT-iresTK and 8 μg/ml polybrene. Next day, the medium was replaced with fresh fibroblast medium. Three weeks post-infection, infected BJ fibroblasts were collected for RNA extraction and TERT expression was examined by qRT-PCR as described below.

### CRISPR/Cas9-Mediated Genome Editing

To generate the CBiPS5 NKX2.5^GFP^ reporter line we used CRISPR/Cas9-targeted genome editing.

**FIGURE 1:**
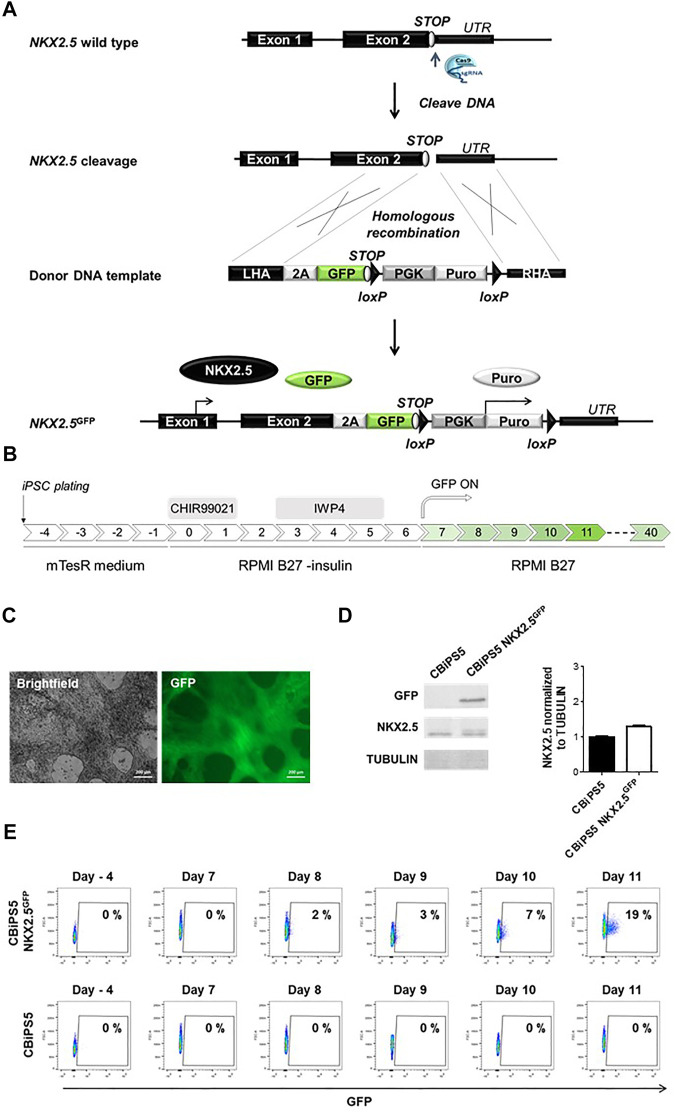
Establishment of NKX2.5^GFP^ hiPSC using CRISPR-Cas9. **(A)** Scheme of the strategy used for the generation of GFP KI at the NKX2.5 locus. **(B)** Schematic representation of the protocol used to induce CBiPS5 NKX2.5^GFP^ cells to cardiac lineages. **(C)** Brightfield and GFP images obtained from Clone 31 of CBiPS5 NKX2.5^GFP^ cells at day 17 of cardiac differentiation under *in vivo* fluorescence microscope. Scale bars, 200 μm. **(D)** Western blot of GFP and NKX2.5 proteins in CM derived from CBiPS5 NKX2.5^GFP^ and parental hiPSC line at day 20 of cardiac differentiation (left panel). Quantification of NKX2.5 protein level of both cell lines normalized to Tubulin is represented (right panel). **(E)** Dot plot diagrams of GFP^+^ cells obtained by flow cytometry at days −4, 7, 8, 9, 10 and 11 of cardiac differentiation in the CBiPS5 NKX2.5^GFP^ and CBiPS5 parental line.

The donor DNA template (LHA-2A-GFP-LoxP-PGK-Puro-LoxP-RHA, Figure 1A) was cloned in a pGEMT vector (Promega) and contained: 1) a Left Homologous Arm (LHA), comprising the 650 base pair (bp) DNA sequence homologous to the coding sequence of NKX2.5 in Exon 2, upstream of the cleavage site without the STOP codon (PCR1); 2) a 2A-GFP-SV40 pA sequence, in frame with the NKX2.5 gene (PCR2); 3) a Puromycin (Puro) resistance cassette regulated by the Phosphoglycerate kinase 1 (PGK) promoter flanked by LoxP sites (LoxP-PGK-Puro-LoXP, PCR4); and 4) a Right Homologous Arm (RHA), comprising the 600 bp DNA sequence homologous to the untranslated region (UTR) of NKX2.5 in Exon 2 located downstream of the cleavage site (PCR5). The DNA fragments of LHA and RHA were PCR amplified from genomic DNA extracted from HFF1 human fibroblasts (ATCC SCRC-1041). The 2A-GFP and LoxP-PGK-Puro-LoxP sequences were PCR amplified from pGEMT.mAgxt.int1. LHA.2A.GFP-RHA and pGEMT.mAgxt.int1. LHA-CoE2EII-puroTK-RHA vectors, respectively, previously generated in the laboratory. These five PCR products were joined using the restriction enzymes described in Supplementary Table S1 to create the final donor DNA template.

Three different sgRNAs targeting NKX2.5 gene were individually cloned into the pSpCas9(BB)-2A-GFP (pX458) vector. (Ran et al., 2013). pX458 was a gift from Feng Zhang (Addgene plasmid # 48,138; http://n2t.net/addgene:48,138; RRID:Addgene_48,138). This vector contains a *S. pyogenes* Cas9 sequence fused to a 2A-GFP sequence and has a cloning site for sgRNA. Cleavage efficiency was analysed in HFF-1 fibroblasts transduced with each sgRNA encoding vector using the Amaxa Nucleofector Kit R (Lonza), X-005 program, with three pulses. Briefly, fibroblasts were harvested using TrypLE for 10 min at 37°C and one million cells per condition were separated and centrifuged. The supernatant was removed and the cell pellet was resuspended in 100 µL of Nucleofector Solution. Then, 5 µg of each sgRNA containing pX458 plasmid were added to 100 µL of cell suspension. Immediately after nucleofection, fibroblasts were transferred onto a gelatine-coated 6-well plate with fibroblast medium (without Pen-Strep for the first 3 days). Transduction efficiency was determined by the percentage of GFP expressing cells 24 h post-nucleofection by flow cytometry. Next, gDNA was extracted from fibroblasts 72 h after nucleofection and a DNA region containing the targeted region was PCR amplified (PCR6) and sequenced by the Sanger method using a specifically designed primer (SeqTIDE NKX2.5) that annealed at 200 nucleotides downstream of the expected cleavage site. The cleavage efficiency of each sgRNA was calculated with the TIDE algorithm (https://tide.nki.nl/).

BJ fibroblasts and CBiPS5 cells were transduced with a Cas9-sgRNA2 ribonucleoprotein (RNP) complex and a linearized donor DNA template using the Neon Transfection System Kit (ThermoFisher Scientific). Briefly, tracrRNA and crRNA2 (Integrated DNA Technologies, IDT) were assembled in a 1:1 M ratio by incubation at 60°C for 10 min and cooled to RT on the bench. Next, Cas9 nuclease protein (IDT) was assembled with synthetic tracr/crRNA2 (sgRNA2) using 1.5 µg of Cas9 nuclease per 1 µg of synthetic sgRNA2 by incubation of the mix for 15 min at RT. Approximately 100,000 BJ fibroblasts and CBiPS5 cells were nucleofected using 0.55 µL (approximately 2.5 µg) of RNP complex and 1 µL (approximately 0.25 µg) of donor DNA. Pulse conditions indicated by the manufacturer for these types of cells were set in a Neon^®^ Device (BJ fibroblasts: 1400 V, 20 ms, 2 pulses; CBiPS5 cells: 1100 V, 30 ms and one pulse). Nucleofected BJ fibroblasts were transferred onto gelatine-coated 6-well plates containing fibroblast medium (without Pen-Strep for the first 3 days). Five days after nucleofection, cells were treated with 1 μg/ml Puromycin and were clonally expanded by plating at a very low density (900 cells/cm^2^) onto gelatine-coated 10-cm plates. Individual colonies were selected using glass cylinders, transferred into a 96-well plate, and expanded. On the other hand, nucleofected CBiPS5 cells were transferred onto Matrigel-coated 6-well plates and cultured in mTesR medium. Five days after nucleofection, cells were treated with 1 μg/ml Puromycin and were clonally expanded by plating at a very low density (850 cells/cm^2^) onto Matrigel-coated plates and cultured in mTeSR medium supplemented with 10% CloneR™ supplement (STEMCELL Technologies) for 24 h. Discrete colonies were manually selected, transferred onto a 12-well plate and expanded.

The correct insertion of donor DNA at the NKX2.5 locus was verified by PCR of gDNA extracted from individual clones (PCR7) and Sanger sequencing using a specifically designed primer (On-target KI). To determine whether the insertion was monoallelic, we performed a PCR amplification of the gDNA with primers that annealed upstream and downstream of the KI sequence (PCR8).

All the primers used for donor DNA template construct generation are described in Supplementary Table S1.

### Differentiation of hiPSC Into CM

To differentiate human hiPSC into cardiac lineages, we used a well-defined protocol*.* (Lian et al., 2012; Lian et al., 2013). Briefly, hiPSC were plated at day -4 of differentiation on Matrigel GFR diluted at 1:100 at 125,000 cells/cm2 and were cultured in mTESR medium (STEMCELL Technologies) supplemented with Pen-Strep, which was changed daily until day 0. From day 0 to day 7 cells were cultured in RPMI medium (Lonza) supplemented with B27 minus insulin and Pen-Strep (both from Gibco). At day 0 of differentiation, cells were treated with 12 µM CHIR99021 (Axon Medchem) for 24 h. On day 3, cells were treated with 5 µM IWP4 (STEMCELL technologies) for 48 h. From day 7 of differentiation onwards, cells were maintained in RPMI medium supplemented with complete B27 (Gibco) and Pen-Strep, and the medium was changed every 3 days.

CM were enriched by two rounds of metabolic selection where indicated. Briefly, on day 11 and 16, the medium was transiently changed to RPMI medium without Glucose (Gibco) supplemented with complete B27, Pen-Strep and 5 mM l-lactic acid (Sigma-Aldrich) for 72 h. From day 14–16, the resting period, cells were cultured with regular differentiation medium.

### Differentiation of hiPSC Into cFib

To differentiate human hiPSC into cFib, we used the protocol described by (Zhang et al., 2019a)*.* Briefly, the first steps of this protocol were the same as those in the cardiac differentiation assay described above, and at day 2.75 of differentiation the medium was switched to cardiac fibroblast basal medium (CFBM, Supplementary Table S2) supplemented with 75 ng/ml bFGF (Peprotech). The medium was changed every other day. On day 20, cells were harvested using TrypLE Express reagent and replated as single cells on dishes precoated with gelatin at a density of 30,000 cells/cm^2^ in FibroGRO medium (SCMF001, Millipore) supplemented with 2% of FBS (Gibco). The medium was changed every other day and cells were passaged every 4–6 days at a cell density of 10,000 cells/cm^2^.

### Gene Expression Analyses by Quantitative Real Time Polymerase Chain Reaction (qRT-PCR)

RNA was isolated using the Maxwell 16 LEV simplyRNA Cells Kit (Promega), and first-strand cDNA was synthesized using the PrimeScript RT reagent Kit (Takara) according to the manufacturer’s recommended protocol. Real-time qPCR was performed on a QuantStudio three or five Real Time PCR Systems (Thermo Fisher Scientific) with PowerUp SYBR Green Master Mix 2X (Applied Biosystems) using 2 ng of cDNA per reaction. The following PCR conditions were established: a first stage at 50°C for 2 min, a second stage at 95°C for 10 min, a third stage consisting of 40 cycles of 95°C for 15 s and 60°C for 1 min, and a fourth stage at 95°C for 15 s, 60°C for 1 min and 95°C for 15 s. All quantifications were normalized to endogenous controls (*GAPDH* and *CYCLOPHILIN*).

Only in the case of COL1A1, LOX and FN1 fibroblast activation marker expression analyses, was first-strand cDNA synthesized using High-Capacity cDNA Reverse Transcription Kit (Applied Biosystem) and qRT-PCR performed on AriaMx Real-Time PCR System (Agilent Technologies) with TaqMan Fast Advanced Master Mix (Applied Biosystems) using 1 ng of cDNA per reaction. The following PCR conditions were used: a first stage at 95°C for 2 min and second stage consisting of 40 cycles of 95°C for 1 s and 60 °C for 20 s. All quantifications were normalized to RNA18S5.

Gene expression data were analyzed in terms of normalized quantification expressed as 2^−ΔCt^ or relative quantification expressed as fold change difference using the 2^−ΔΔCt^ method, where indicated. The list of primers used for qRT-PCR analyses are described in Supplementary Tables S3, 4.

### Immunofluorescence

GFP^−^ and GFP^+^ cells sorted at day 11 of differentiation were plated on a 24 well-plate containing microscope cover glasses (Paul Marienfeld GmbH & Co. KG) coated with Matrigel diluted 1:100 at a density of 30,000 cells per cover glass and maintained for 24 h in RPMI B27 medium, supplemented with 5 µM ROCK inhibitor.

On the other hand, NKX2.5^GFP^-cFib at passage five were dissociated with TrypLE express reagent and plated on a 24 well-plate containing microscope cover glasses (Paul Marienfeld GmbH & Co. KG) coated with 0.1% gelatin at a density of 50,000 cells/cm^2^. cFib were cultured in FibroGRO medium supplemented with 2% of FBS for 3 days. In addition, α Smooth Muscle Actin (SMA) activation marker expression was analyzed in cFib after 48 h treatment with TGF-β (detailed below).

Cells were fixed with 4% paraformaldehyde (PFA, Sigma-Aldrich) for 15 min at RT, and washed three times with PBS. After fixation, cells were blocked and permeabilized with blocking solution (PBS containing 10% goat serum, 1% BSA and 0.1% Triton X-100 Sigma-Aldrich) for 30 min at RT. Cells were incubated overnight at 4°C with primary antibodies diluted in blocking solution. Cells were washed with PBS at RT (10 min each wash, three times) and the secondary antibodies diluted in the blocking solution were incubated at RT for 45–60 min in the dark. After washing immunostained cells with PBS at RT (10 min each wash, three times), preparations were mounted in Faramount fluorescent mounting medium containing 4,6-diamidino-2-phenylindole (DAPI; 100 ng/ml; Molecular Probes) to detect all nuclei and analyzed in an automated fluorescence microscope (Zeiss Axio Imager M1). The antibodies used for the immunofluorescence analyses are detailed in Supplementary Table S5.

### Flow Cytometry and Fluorescence-Activated Cell Sorting (FACS) Analyses

CBiPS5 NKX2.5^GFP^ cells at day 11 of cardiac differentiation were dissociated with TrypLE dissociation reagent (Gibco) as described above and filtered through a 35 µm strainer snap cap in a 5 ml test tube (Falcon).

For fluorescence-activated cell sorting (FACS), live cells were resuspended at 10^7^ cells/ml in Sorting Buffer [PBS without Ca^2+^ nor Mg^2+^, 1 mM EDTA, 0.5% FBS and 1% Pen Strep and sorted on a BD FACSAria II instrument (BD Biosciences)]. Flow cytometry gates were set to minimize cross-contamination by neighboring subpopulations. For experiments of genetic and functional characterization, collected GFP^+^ and GFP^−^ cell fractions were replated onto Matrigel-coated 12-well or 96-well plates in RPMI B27 medium, supplemented with 5 µM ROCK inhibitor during the first 24 h after plating.

One hundred thousand cells from NKX2.5^GFP^-cFib cell cultures at passage 4 and parental non-differentiated CBiPS5 NKX2.5^GFP^ cells were immunostained with 100 ng of SSEA4-APC antibody (FAB1435A, R&D systems). Flow cytometry data acquisition was performed with a BD FACSCanto II (BD Biosciences) cytometer.

BD FACSDiva v6.1.3 software (BD Biosciences) was used for data file collection, and data analyses were performed with the FlowJo v10 (Tree Star Inc.) software package.

### Immunobloting

CBiPS5 NKX2.5^GFP^ and parental non-modified CBiPS5 cells were differentiated into cardiac lineage, and at day 20 of differentiation, after two rounds of metabolic selection as described above, cardiomyocytes were detached with TrypLE dissociation reagent and collected for protein extraction. Cells were washed once with cold PBS (Gibco) and incubated with 0.5–1 ml of lysis buffer [1% Triton, 50 mM Tris-HCl, 150 mM NaCl, 10 mM NaF, 1 mM Sodium Orthovanadate and cOmplete™ Mini protease inhibitor cocktail (Roche)] for 30 min on ice. Protein concentration was quantified with the Pierce BCA protein assay (Thermo Scientific), following the manufacturer’s instructions. Protein samples were denatured by incubation for 5 min at 95°C in 5x loading buffer (50% Glycerol, 250 mM Tris-HCl, 10% SDS, 12,5% 2-mercaptoethanol, Bromophenol blue).

Each sample was subjected to electrophoresis in 1.5 mm thick 10% SDS-PAGE gel (NKX2.5) or 12% SDS-PAGE gel (GFP) using the Mini-PROTEAN Tetra Handcast System (Bio-Rad). After electrophoresis, proteins were transferred to a nitrocellulose membrane (Bio-Rad) using the Wet/Tank Blotting System (Bio-Rad) at 350–400 mA for 2 h and were blocked for 1 h at RT in PBST (PBS, 0.05% Tween 20) supplemented with 5% of non-fat dry milk. Then, membranes were incubated O/N at 4°C with goat NKX2.5 antibody (Santa Cruz, sc-8697, 1:1,000), or goat GFP antibody (Novusbio, GFP-1010, 1:2,000) diluted in blocking solution. After three washes with PBST, membranes were incubated with rabbit anti-goat IgG AP (Sigma-Aldrich, A4062, 1:50,000) or goat anti-mouse IgG AP (Sigma-Aldrich, A9316, 1:50,000) for 1 h at RT. Membranes were washed three times with PBST and treated for 5 min with a mixture of Tropix CSPD Ready-to-use and Tropix Nitro-Block II (Applied Biosystems) and visualized by chemiluminescence using the ChemiDoc Touch Imaging System (BioRad). Then, the membrane was incubated with mouse β-tubulin antibody (Sigma-Aldrich, T4026, 1:100) followed by incubation for 1 h with goat anti-mouse IgG AP, and this protein was used as a loading control. Protein band density was quantified using ImageJ (NIH).

### Electrophysiological Activity

Microscopic optical mapping analyses were performed in NKX2.5-GFP^+^-derived CM (NKX2.5-GFP^+^-CM). Briefly, NKX2.5-GFP + cells were sorted at day 11 of cardiac differentiation, plated, subjected to metabolic selection, and maintained until day 30 as described above. Electrical activity was measured by intracellular Ca^2+^ propagation transient imaging. Stimulation-induced concentration of intracellular Ca^2+^ changes was measured in cells using the Ca^2+^-sensitive fluorescent ratiometric dye Fura-2 acetoxymethyl ester (Fura-2 AM, TEFLabs, Austin, TX) such that when Ca^2+^ is bound it emits at 340 nm, whereas when it is unbound, it emits at 380 nm. Fluorescence was recorded with an EMCCD camera (Evolve-128: 128 × 128 imaging pixels, 24 × 24-μm pixels, 16 bits; Photometrics, Tucson, AZ, United States), with a custom multiband-emission filter (ET585/50-800/200 M; Chroma Technology) placed in front of a high-speed camera lens (DO-2595; Navitar Inc., Rochester, United States). This camera records images from 340 to 380 nm filters and combines the readouts of both filters to create a 340/380 ratio, which correlates with the amount of intracellular calcium. Acquired signals were processed and quantified as described in previous publications (Gómez-Cid et al., 2020) using a MATLAB software-based interface.

Patch-clamp assays were conducted in NKX2.5-GFP^+^-CM at days 30–35 of differentiation. CM were treated with TrypLE express reagent (Gibco), and single cells were seeded and sedimented on a 0.3 ml perfusion camera (Warner Instruments) mounted on an inverted microscope (DMIL, Leica) for patch-clamp recordings. These recordings were made using an Axon Axopatch 200B Patch-Clamp amplifier (Molecular Devices). Glass pipettes were prepared using thin-wall borosilicate glass (Sutter Instrument) with a micropipette puller (PP-830, Narishige) and had a tip resistance of 1–3 MΩ when filled with a pipette solution containing (in mM): K-Aspartate 80, KCl 50, NaCl 5, KH_2_PO_4_ 10, MgSO_4_ 1, Na_2_-ATP 3, HEPES 5, and EGTA 10; pH was adjusted to 7.2 with KOH. Action potentials (APs) were recorded from CMs superfused with Tyrode solution at RT. The Tyrode solution containing (in mM): NaCl 130, KCl 4, CaCl_2_ 1.8, MgCl_2_ 1, HEPES 10, and glucose 11; pH was adjusted to 7.4 with NaOH. Data were digitized with an AC/DC converter Digidata 1440A (Molecular Devices) at 1.0 kHz and acquired using the Clampex program of the pClamp 10.2 software (Molecular Devices). Data were analyzed using the Clamfit program of the pClamp 10.2 software (Molecular Devices). Cardiomyocyte subtypes were classified using a ratio of (APD10-20/APD60-70). An AP with a ratio <1.5 was classified as the atrial-like subtype, whereas a value of >1.5 was categorized as the ventricular-like cardiomyocyte (modified from Ma *et al.* (Ma et al., 2011)).

### Fibroblast Activation Assays

In these assays, cFib derived from two different patients were included (Patient one cFib and Patient 2 cFib) as a reference.

NKX2.5^GFP^-cFib and patient-derived fibroblasts were harvested and plated at a density of 50,000 cells/cm^2^ on 6-well plates in their corresponding culture medium. When fibroblasts reached 70% confluence, cells were serum-deprived for 8 h and, in the case of patient-derived cFib, bFGF the concentration was reduced from 10 ng/ml to 0.5 ng/ml. Some wells were treated with 10 ng/ml of TGF-β (R&D systems) and non-treated wells were used as controls. After 24 h or 48 h, cFib activation marker expression was analyzed, by qPCR (COL1A, LOX, FN1, SMA) or immunofluorescence (SMA), respectively, as indicated above.

### Statistical Analyses

Statistical analyses were performed using GraphPad Prism software. In the case of NKX2.5-GFP^+^ cell gene expression analyses, data of technical triplicates (median presented) of two independent biological replicates (Exp1 and Exp2) are shown. Statistical comparison between GFP^−^ and GFP^+^ groups was performed using via Nested-t tests. In the case of fibroblast activation assays, data are presented as mean ± SD of technical triplicates in the case of three patient-derived samples and mean ± SD of three biological replicates in the case of NKX2.5-GFP^+^-cFib. Statistical comparison between treated and non-treated samples was carried out via 2WAY ANOVA test. A *p* < 0.001 and *p* < 0.01 is defined as very significant, *p* < 0.05 as significant, and *p >* 0.05 as non-significant (ns).

## Results

### Generation of Human NKX2.5^GFP^ hiPSCs Using CRISPR-Cas9 System

We first established a NKX2.5-GFP knock-in human hiPSC reporter cell line. To generate this cell line, we designed a knock-in strategy to avoid perturbation of the reading frame of NKX2.5 and therefore loss-of-function which could affect the biology of cardiovascular progenitors and CM (Figure 1A). In this strategy, a 2A-GFP sequence was placed downstream of the endogenous NKX2.5 coding sequence.

We used CRISPR-Cas9 technology to mediate directed insertion of donor DNA HDR (homologous Directed Repair). We designed and tested three different sgRNAs targeting the NKX2.5 gene (Supplementary Figure S1A). The sgRNA2 was selected for further experiments since it presented the highest cleavage efficiency, 49% of the transduced cells (Supplementary Figure S1B).

Next, we transduced a human hiPSC line previously established in our laboratory, CBiPS1sv-4F-5 (Arellano-Viera et al., 2019) (abbreviated here as CBiPS5) with the ribonucleoprotein (Cas9-sgRNA2 complex) and the donor DNA, and nucleofected cells were treated with 1 μg/ml Puromycin to select CBiPS5 cells with donor DNA integrated in their genome. Clones from transduced and puromycin resistant hiPSCs were established, and 7 out of 72 analyzed clones, about 10% of the clones, presented GFP at the NKX2.5 locus (Supplementary Figure S1C). The NKX2.5-2A-GFP sequence was verified by the Sanger sequencing method, and PCR results showed that the insertion of the donor DNA was monoallelic in these 7 clones (Supplementary Figure S1D).

To check if the reporter system recapitulates *NKX2.5* expression in CBiPS5 NKX2.5^GFP^ clones, we differentiated these cells into cardiac lineages using a well-established protocol (Lian et al., 2013; Lian et al., 2012) based on the biphasic modulation of the WNT signaling pathway (Figure 1B) in which activation of canonical WNT by the treatment with high concentrations of a GSK3β inhibitor (CHIR99021) directs mesoderm commitment of hiPSCs, and the subsequent inhibition of WNT signaling by the inactivation of Porcupine with IWP4 and induces cardiac progenitor differentiation into CM. We first analyzed GFP expression by *in vivo* fluorescence microscopy and found that it was absent in the pluripotency stage. However, it became visible in all clones from day 9–10 of differentiation onwards (data not shown), in accordance with the reported timing of NKX2.5 activation using this protocol. (Zhang et al., 2019a; Zhang et al., 2019b). Clone 31 was selected to perform an exhaustive characterization (Figure 1C, Supplementary Video S1, 2).

In order to verify that the NKX2.5 protein and function were not disrupted in CBiPS5 NKX2.5^GFP^ cells after genome editing, we performed a western blot assay using protein samples extracted from both non-transduced parental hiPSC line cardiomyocytes derived from CBiPS5 NKX2.5^GFP^ cells at day 20 of cardiac differentiation, after metabolic selection of CM by lactate treatment. We observed that the protein level of NKX2.5 was comparable between CBiPS5 NKX2.5^GFP^ and unmodified CBiPS5 cells, and, as expected, GFP protein was only expressed in CBiPS5 NKX2.5^GFP^ cells (Figure 1D **)**. Additionally, to assess NKX2.5 functionality, we analyzed the expression of several NKX2.5 target genes identified in CM derived from human PSCs (Nakashima et al., 2009) and found that the expression of these genes was very similar between reporter and parental hiPSC lines suggesting preservation of NKX2.5 transcriptional regulatory function (Supplementary Figure S2A).

GFP expression was detected by flow cytometry from day 8 of differentiation onwards and the percentage of GFP-positive (GFP^+^) cells progressively increased until day 11 of differentiation (Figure 1E). However, mRNA expression of GFP was detected by qRT-PCR 1 day earlier, from day 7 of cardiac differentiation onwards, and importantly, the expression kinetics of GFP mirrored the expression of NKX2.5 (Supplementary Figure S2B). Moreover, the NKX2.5 and GFP proteins were co-expressed in FACS-sorted GFP^+^ cells (Supplementary Figure S2C).

### NKX2.5-GFP^+^ Cells Differentiate Mainly Into Ventricular CM

**FIGURE 2:**
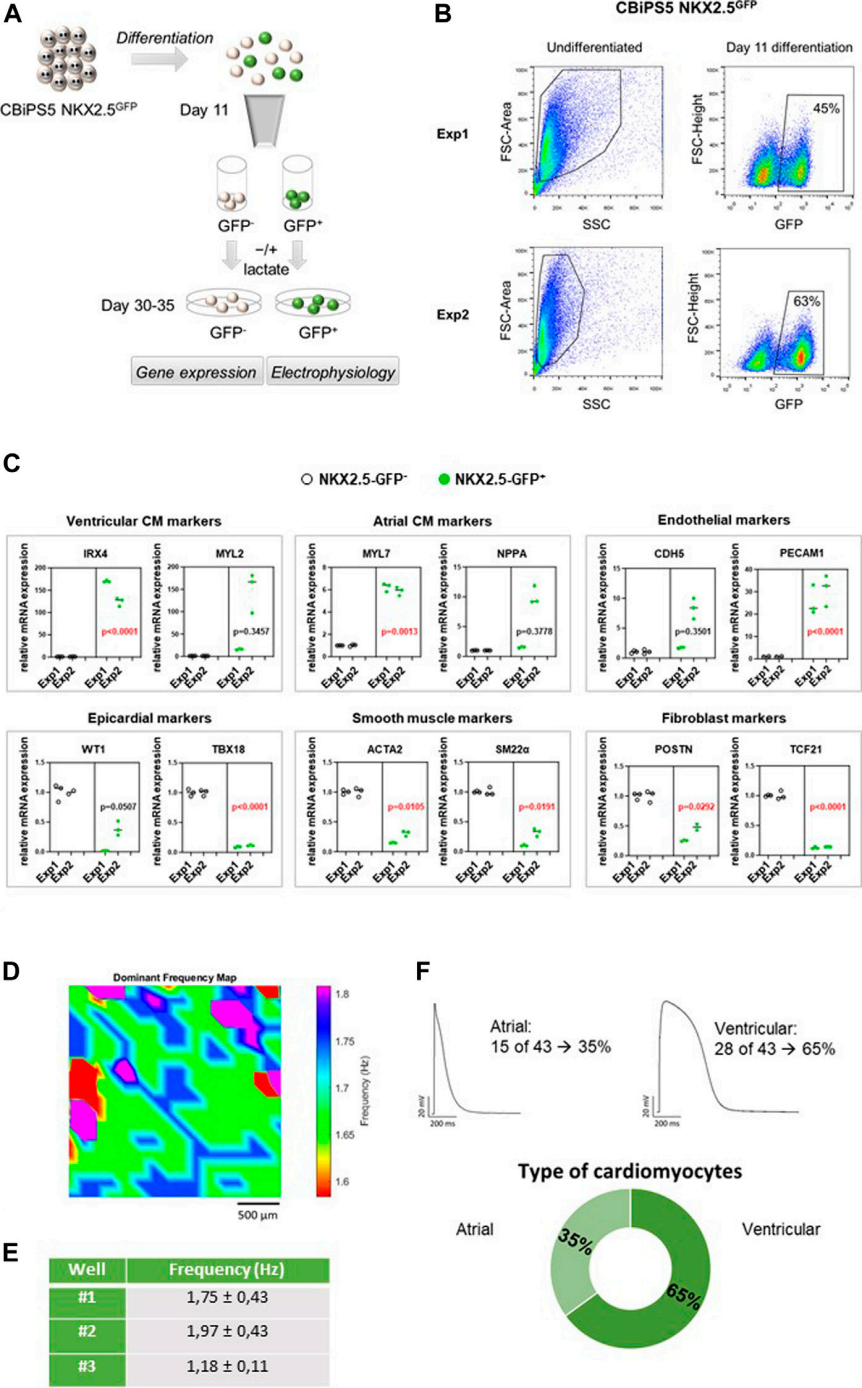
NKX2.5-GFP^+^ cell characterization. **(A)** Representation of the analyses performed in GFP^+^ and GFP^−^ cells sorted at day 11 of differentiation. **(B)** Dot plot diagram of GFP^+^ cells obtained by flow cytometry analysis of pre-sorted CBiPS5 NKX2.5^GFP^ cells at day 11 of cardiac differentiation in two different assays. **(C)** Gene expression analysis of specific cardiac lineage markers in NKX2.5-GFP^+^ (green circles) and NKX2.5-GFP^-^ (black circles) cell derivatives at day 30 of cardiac differentiation. Two biological replicates (Exp1 and Exp2), three technical replicates in each. Median of three technical replicates in two biological replicates (Exp1 and Exp2) are represented; the *p* value is annotated in each graph, GFP^−^ group vs GFP^+^ group using Nested-t tests. **(D)** Activation frequency map for well #1 is shown. Baseline recordings acquired from 10 to 30 frames per second in a field of view of 2.5 mm **(E)** Activation frequency registrations (Hz) obtained by optical mapping of NKX2.5-GFP^+^ cells-derived CM in three independent wells. **(F)** Patch-clamp recordings in GFP^+^ cells-derived CM. Atrial and ventricular-like action potentials and percentages of each cardiomyocyte subtype are represented.

It is known that NKX2-5^+^ cardiovascular progenitors differentiate into atrial and predominantly ventricular but not nodal-like CM. (Zhang et al., 2019b; Birket et al., 2015). To reveal the differentiation potential of NKX2.5-GFP^+^ cells, CBiPS5 NKX2.5^GFP^ cells were induced into cardiac lineages and the expression of cell lineage-specific markers was investigated in GFP^+^ and GFP^−^ cell derivatives. To this end, these cell populations were sorted by FACS at day 11 of cardiac differentiation, replated and maintained until day 30–35 (Figure 2A). As expected, the NKX2.5 gene was highly expressed in the GFP^+^ cell population, both on day 11 (Supplementary Figure S2D) and day 30 (Supplementary Figure S2D) of differentiation, reaffirming the reliability of the reporter. On day 1, MYH6 and PLN pan CM markers were robustly expressed in NKX2.5-GFP^+^ cells and differentiated progeny derivatives when compared to GFP^−^ cell populations; in contrast, TBX18 and WT1 epicardial markers were increased in GFP^−^ cells and their derivatives (Supplementary Figure S2D). At day 30 of differentiation, we observed that ventricular cardiomyocyte (IRX4, MYL2), atrial CM (MYL7 and NPPA) and endothelial (PECAM1 and CDH5) specific markers were upregulated in GFP^+^ cell derivatives whereas nodal-like CM (TBX3, SHOX2), epicardial (WT1, TBX18), smooth muscle (ACTA2, SM22α) and fibroblast (TCF21, VIM) specific genes were overexpressed in GFP^−^ cell derivatives (Figure 2C). These results were in agreement with previously reported observations. (Birket et al., 2015; Zhang et al., 2019b).

To study the electrophysiological properties of GFP^+^ cells-derived CM, microscopic optical mapping and patch-clamp assays were performed. We analyzed the functionality of CM derived from GFP^+^ cells at day 30 of cardiac differentiation using optical mapping that measures electrical activity by intracellular Ca^2+^ propagation imaging. To this end, cells were stained in Tyrode solution with Fura-2 AM and activation frequency was recorded. We analyzed three independent wells that showed spontaneous beating. Samples showed infusion of the die and propagation of the electrical impulse was observed (Figure 2D, Supplementary Video S3). Calcium activity was mapped and overall mean activation frequency of the three wells registered was approximately 1.63 ± 0.65 Hz (Figure 2E). To more precisely characterize in which subtype of CM the GFP^+^ cells were differentiated, patch-clamp recordings in single GFP^+^ cells-derived CM were carried out at days 30–35 of differentiation. Resting potential, action potential amplitude and durations at 10, 20, 60, 70 and 90% of repolarization were measured. CM subtypes were determined attending to APD10-20/APD60-70 ratio. An AP with a ratio <1.5 was classified as the atrial-like subtype, whereas a value of >1.5 was categorized as the ventricular-like CM (Ma et al., 2011). Thus, we determined that 65% of registered cells were ventricular CM and 35% were atrial CM, which represents a 2:1 ratio of ventricular versus atrial CM (Figure 2F). We did not detect any nodal CM. Our results were in line with previous studies. (Birket et al., 2015; Zhang et al., 2019b).

### Generation of Functional NKX2.5^GFP^ Reporter Human Fibroblasts

We first tried to generate a knock-in of GFP at the NKX2.5 locus in BJ neonatal foreskin human fibroblasts, following the same strategy as used for hiPSC. We chose BJ fibroblasts since these fibroblasts, unlike adult fibroblasts, can support the long-term cell culture which is required to generate and establish a reporter cell line. BJ fibroblasts were nucleofected with RNP-sgRNA2 complex and donor DNA, treated with puromycin and plated at clonal dilution. Individual colonies were selected and expanded but, in a few passages, became senescent and could not be analyzed. This occurred repeatedly in multiple experiments. We next tried to immortalize BJ fibroblasts by overexpression of TERT by lentiviral infection with pLOX-TERT-iresTK. (Salmon et al., 2000). Quantitative real time polymerase chain reaction (qRT-PCR) analysis showed a prominent TERT expression (Supplementary Figure S3A). Using this strategy, we were able to establish clones from nucleofected BJ-TERT fibroblasts, and we found that from 32 expanded clones 2 contained an NKX2.5 edited allele (Supplementary Figure S3B). However, we again observed that the proliferation rate of these fibroblasts progressively fell and we were thus unable to establish a reporter BJ cell line.

**FIGURE 3:**
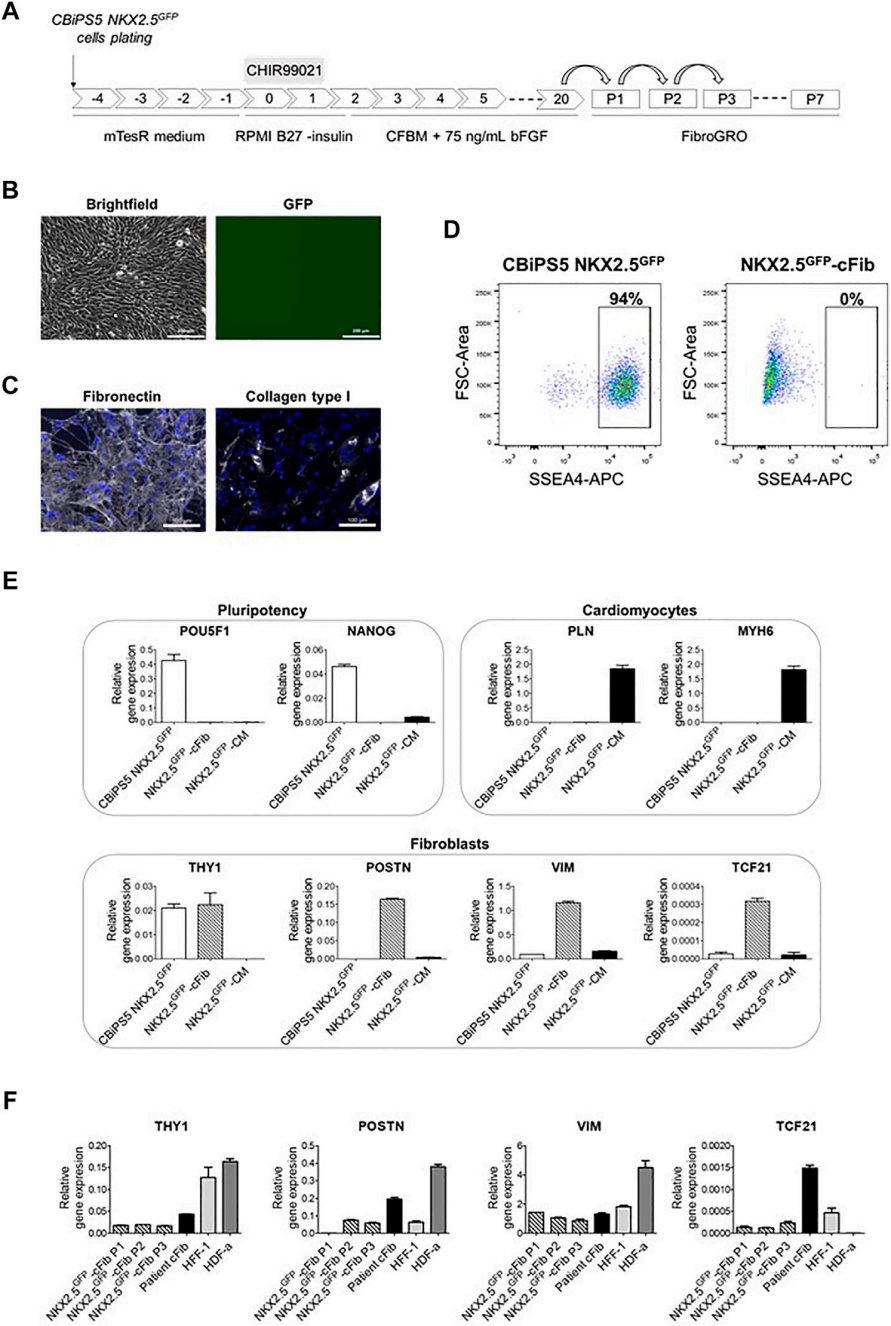
Characterization of NKX2.5^GFP^-cFib. **(A)** Scheme of the fibroblast differentiation protocol used for the generation of NKX2.5^GFP^-cFib derived from the CBiPS5 NKX2.5^GFP^ cell line. **(B)** Brightfield and GFP images obtained from CBiPS5 NKX2.5^GFP^-cFib under *in vivo* fluorescence microscopy. Scale bars, 200 µm **(C)** Immunostaining analysis of fibronectin (in white) and collagen type I (in white) in NKX2.5^GFP^-cFib. Nuclei: DAPI. Scale bars, 100 µm **(D)** Dot plot diagrams of SSEA4^+^ cells obtained by flow cytometry in CBiPS5 NKX2.5^GFP^ cells and NKX2.5^GFP^-cFib. **(E)** Gene expression analysis of pluripotency (OCT4 and NANOG), cardiomyocyte (PLN and MYH6) and fibroblast (THY1, POSTN, VIM and TCF21) specific genes in NKX2.5^GFP^-cFib, CBiPS5 NKX2.5^GFP^ cells and CBiPS5 NKX2.5^GFP^-derived CM (NKX2.5^GFP^-CM). **(F)** Gene expression analysis of fibroblast-specific markers (VIM, POSTN, TCF21 and THY1) in NKX2.5^GFP^-cFib at passages 1, 2 and three and in patient-derived cFib, HFF-1 and HDF-α fibroblast cell lines. Mean ± SD represented of three technical replicates is represented.

Our next approach was to obtain human NKX2.5^GFP^ fibroblasts through directed differentiation of CBiPS5 NKX2.5^GFP^ cells. To this end, we used a recently described method in hiPSC for the generation of human cFib derived from cardiac progenitors analogous to SHF, (Zhang et al., 2019a), which is based on the treatment of cells with a high dose of the CHIR99021 GSK3β inhibitor to form cardiac mesoderm, as in a cardiac differentiation protocol, followed by stimulation with a high concentration of bFGF to induce cFib (Figure 3A). Then, NKX2.5^GFP^-cFib were maintained, frozen in the first passages and expanded for characterization.

NKX2.5^GFP^-cFib derived from CBiPS5 NKX2.5^GFP^ cells had a spindle-like morphology and did not present basal GFP expression (Figure 3B). These fibroblasts bore GFP at the NKX2.5 locus as expected (Supplementary Figure S3C). The extracellular matrix proteins fibronectin and collagen type I were expressed in NKX2.5^GFP^-cFib, as detected by immunocytochemistry (Figure 3C). Next, we examined the expression of pluripotency markers. Flow cytometric analysis showed that NKX2.5^GFP^-cFib did not express the cell surface antigen SSEA4 (Figure 3D), and the qRT-PCR analyses showed that POU5F1 and NANOG transcription factor expression was completely downregulated when compared with parental CBiPS5 NKX2.5^GFP^ cells, as analyzed by qRT-PCR (Figure 3E). On the other hand, PLN and MYH6 CM-specific gene expression was not detected in NKX2.5^GFP^-cFib when compared with CBiPS5 NKX2.5^GFP^-CM (Figure 3E). In contrast, NKX2.5^GFP^-cFib expressed fibroblast-specific markers such as THY1 (cell surface marker for distinct fibroblast populations), POSTN (expressed in cFib during embryogenesis (Snider et al., 2009) and increeased in adult cFib upon cardiac injury (Kanisicak et al., 2016; Fu et al., 2018)), VIM (an intermediate filament that modulates fibroblast traction force (Ho Thanh et al., 2021)) and TCF21 (a transcription factor required for cFib development (Acharya et al., 2012; Kanisicak et al., 2016)). We investigated the expression of these fibroblast-specific markers in NKX2.5^GFP^-cFib during the first three passages. In this study, we included fibroblast lines derived from different sources such as patient-derived cFib obtained from atrial biopsy (patient cFib), an HFF-1 human foreskin fibroblast cell line and an HDF-α human dermal fibroblast cell line. We found that all types of fibroblasts, except for HDF-α in the case of TFC21, expressed VIM, POSTN, TCF21 and THY1 despite expressing different levels depending on the origin of the fibroblast (Figure 3F). Interestingly, even NKX2.5^GFP^-cFib at the first passage expressed these markers, except for POSTN, which increased from the second passage onwards.

**FIGURE 4:**
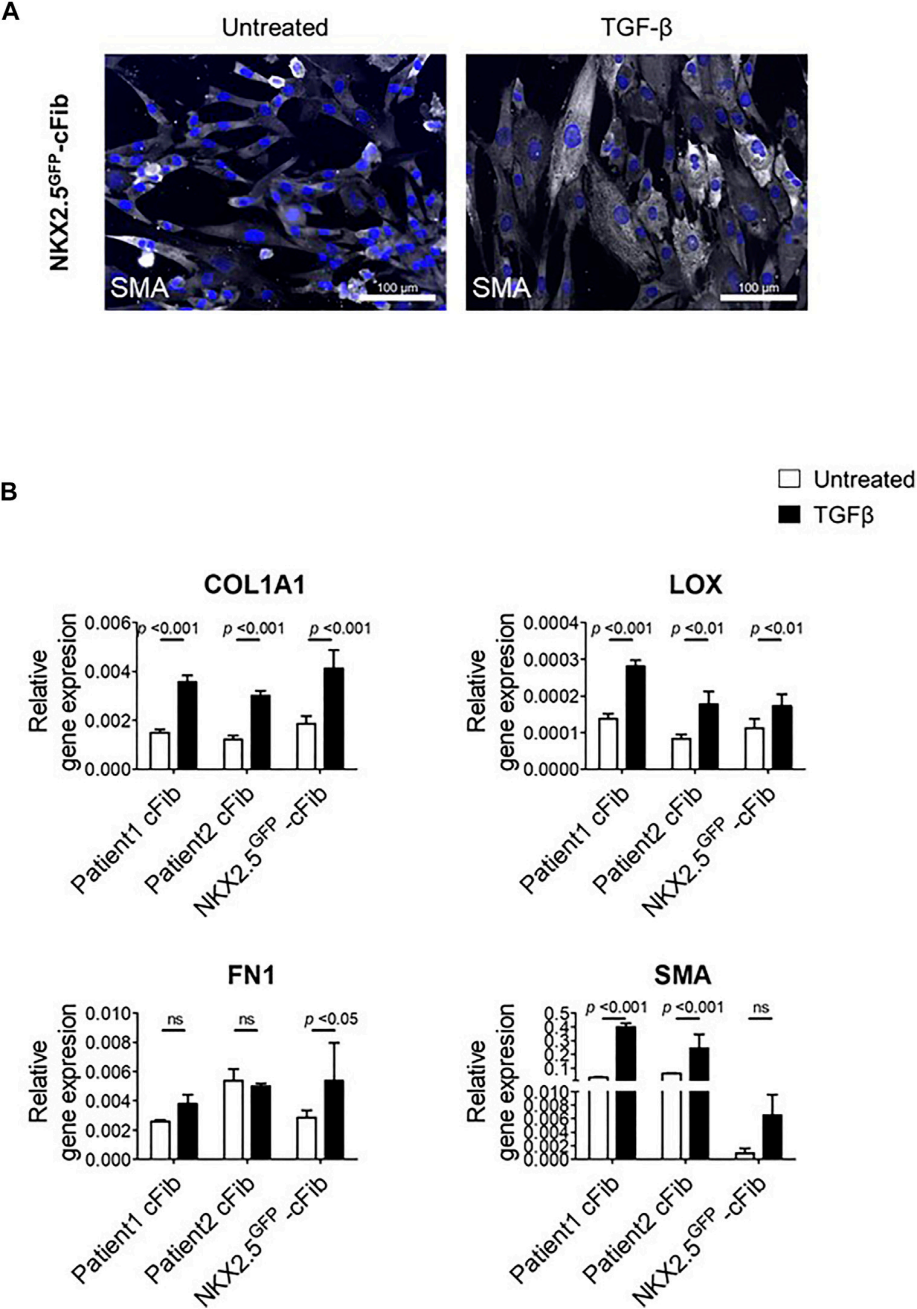
Fibroblast activation assays in NKX2.5^GFP^-cFib. **(A)** Immunostaining analysis of αSMA (in white) in NKX2.5^GFP^-cFib non-treated and treated with 10 ng/ml TGFβ for 48 h. Nuclei: DAPI. Scale bars, 100 µm **(B)** Gene expression analysis of activated fibroblast markers (COL1A1, LOX, FN1, SMA) in NKX2.5^GFP^-cFib and cFib derived from two different patients, non-treated (white bars) and treated (black bars) with 10 ng/ml TFGβ for 24 h. Three biological replicates were included in NKX2.5^GFP^-cFib data, and three technical replicates in the case of patient-derived cFib. Mean ± S.D. of each data group are represented; non-treated *vs* treated *p* values are shown using a 2 way ANOVA test.

Finally, we performed a functional assay in order to evaluate NKX2.5^GFP^-cFib activation capacity in response to TGF-β. For this study, we included two patient-derived cFib as controls. TGF-β levels increase substantially after cardiac injury which triggers the transformation of cardiac fibroblasts into myofibroblasts, to enhance the production and secretion of extracellular matrix proteins and subsequently prevent ventricular wall rupture. (Lijnen et al., 2000; Bujak and Frangogiannis, 2007). NKX2.5 GFP-cFib were serum-deprived overnight and stimulated with 10 ng/ml TGF-β. Treated NKX2.5 GFP-cFib acquired a well-spread morphology, clearly noticeable with αSMA immunostaining (Figure 4A). We also analyzed gene expression of several markers known to be increased in activated fibroblasts and a significant increase in the expression of COL1A, LOX and FN1 was detected in NKX2.5 GFP-cFib treated with TGF-β, similar to the activation observed in patient-derived cFib (Figure 4B).

## Discussion

*In vivo* direct cardiac reprogramming has emerged as an attractive therapeutic approach to treat injured hearts through the transformation of cFib into CM. (Qian et al., 2012; Song et al., 2012). Although significant progress has been made in the cardiac reprogramming field in recent years, robust and reproducible protocols are still needed to be set up *in vitro*, mainly in the case of human cells, before this strategy can be translated into the clinic. (López-Muneta et al., 2020; Garry et al., 2021). In this study, we have generated functional human NKX2.5^GFP^ cFib to facilitate screening factor research. In addition, since NKX2.5 is expressed not only in CM but also in earlier cell stages such as cardiovascular progenitors, (Elliott et al., 2011; Den Hartogh et al., 2015), the detection of both cell types would be possible.

We tried to generate reporter fibroblasts directly by nucleofection of these cells with a CRISPR-Cas9 nucleoprotein and donor template. However, even starting with BJ neonatal fibroblasts, which have a greater proliferative capacity than adult fibroblasts, and overexpressing TERT in these fibroblasts, our attempts were unsuccessful, and fibroblasts stopped proliferating, which prevented the generation of reporter fibroblast lines. Since hiPSC have the ability to differentiate into cFib and these are the target cells for *in vivo* cell reprogramming, we decided to generate a human NKX2.5^GFP^ hiPSC line and obtain NKX2.5^GFP^-cFib through directed differentiation. The advantage of using this approach is that hiPSC have unlimited proliferation potential and cFib derived from these cells still retain a high proliferative capacity, (Zhang et al., 2019a), which provides an unlimited supply of cellular models for research in cardiac reprogramming. In addition, the established NKX2.5^GFP^ hiPSC line could also represent a useful platform for screening of inducers of cardiac differentiation.

Thus, we generated NKX2.5-GFP knock-in human hiPSCs. In these reporter hiPSCs, upon directed cardiac differentiation, GFP and NKX2.5 were concomitantly expressed transcriptionally from day 7 onwards although fluorescence was detected first at day 8 by flow cytometry in a small percentage of cells and was visible at day 9–10 under fluorescence microscopy, in line with previous reports. (Zhang et al., 2019a; Zhang et al., 2019b). We showed that GFP reporter faithfully recapitulated the NKX2.5 activity. (Birket et al., 2015; Zhang et al., 2019b). Gene expression and electrophysiological analyses demonstrated that early expressing GFP cells differentiated mostly towards ventricular-like CM and to a lesser extent into atrial-like CM. Other cell lineage specific markers for smooth muscle, fibroblast or epicardial cells, as well as for nodal-like CM, were enriched in non-expressing GFP cells as expected. (Birket et al., 2015; Zhang et al., 2019b).

Next, these NKX2.5^GFP^ hiPSCs were robustly differentiated into functional cFib. It is known that fibroblasts of different developmental origin can be activated after cardiac injury. (Kanisicak et al., 2016; Tallquist and Molkentin, 2017). The protocol used here can efficiently generate human hiPSC-derived cFib from the SHF-derived endocardial cells (Zhang et al., 2019a) that constitute a significant population of cFib in the adult heart. (Tallquist and Molkentin, 2017). However, other protocols to generate epicardium-derived cFib (Zhang et al., 2019c) could be applied. These NKX2.5^GFP^ hiPSCs-derived fibroblasts expressed fibronectin and collagen type I extracellular matrix proteins, and other fibroblast-related markers at the RNA level such as vimentin (VIM), periostin (POSTN), transcription factor 21 (TCF21) or CD90 (THY1). Interestingly, the levels of expression of these genes were similar to patient-derived cFib, except in the case of TCF21. This result might be explained by the fact that patient samples include fibroblasts of different embryonic origin such as epicardial fibroblasts that express high levels of TCF21. On the other hand, pluripotency- and CM-related markers were abrogated in these fibroblast cultures. Importantly, these reporter fibroblasts were able to transform into myofibroblasts in response to TGF-β treatment. We observed that the morphology of cells changed and they became larger SMA^+^ myofibroblasts. In addition, several markers known to be overexpressed in activated fibroblasts (COL1A, LOX and FN1) were induced after TGF-β treatment. Although human hiPSC-derived cFib are considered to have a more embryonic phenotype (Zhang et al., 2019a) and their immaturity could be a concern, we demonstrated here that the expression of several fibroblast-related markers and the induction of profibrotic response was similar in NKX2.5^GFP^-cFib and patient-derived cFib. In addition, the main advantages of using these cells as platforms for direct cardiac reprogramming screenings are that these fibroblasts, unlike other commonly used fibroblasts such as dermal or neonatal fibroblasts, are of cardiac origin, which is interesting since resident cardiac fibroblasts in the human heart will be the main target cell type for *in vivo* direct cardiac reprogramming in the future and, furthermore, these cardiac fibroblasts can be easily obtained through directed differentiation of hiPSC and have a high proliferation capacity which is crucial to obtain an unlimited source of cells and allow the screening of reprogramming factors *in vitro*, in contrast to patient-derived cardiac fibroblasts whose accessibility is restricted, their isolation requires an invasive method and senesce rapidly after few passages *in vitro*.

**FIGURE 5:**
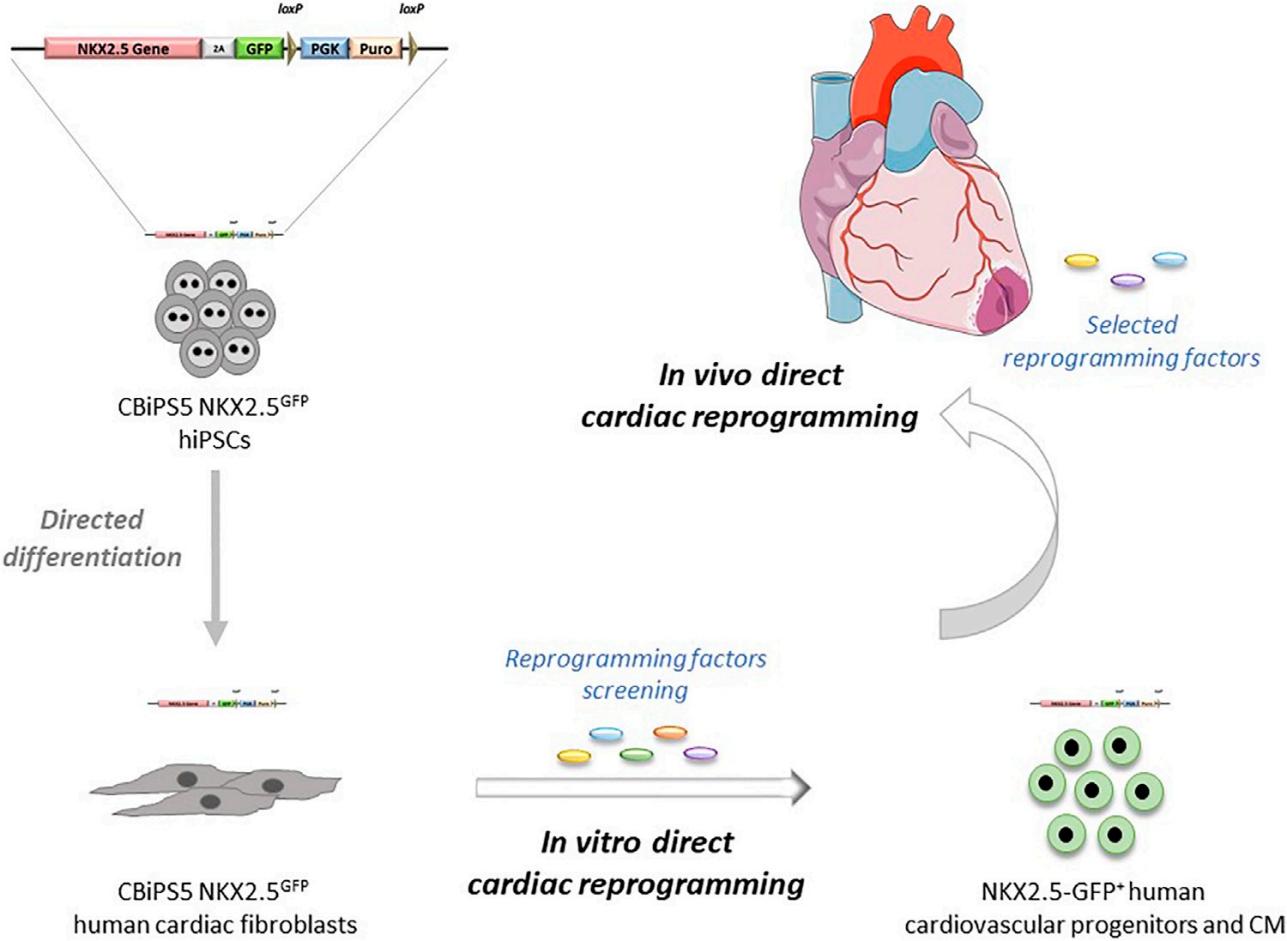
Potential applications of NKX2.5^GFP^ reporter cell lines in direct cardiac reprogramming.

In summary, in this report we have generated a new human NKX2.5^GFP^ hiPSC line by CRISPR-Cas9-mediated homologous recombination in which NKX2.5 expression and function are not affected after the genome editing. The reliability of the reporter to track NKX2.5 expressing cells and the potential of NKX2.5-GFP^+^ cells to give rise to the expected cardiac lineages was demonstrated. Then cardiac fibroblasts were obtained through directed differentiation of NKX2.5^GFP^ hiPSC. These NKX2.5^GFP^ cardiac fibroblasts had the typical fibroblast-like morphology, expressed fibroblast markers, and showed functionality in response to TGF-β stimulation, similar to that found in patient-derived cardiac fibroblasts. Our approach offers an unlimited supply of cellular models for research and advancement of human direct cardiac reprogramming (Figure 5) and we are willing to share these NKX2.5^GFP^ reporter lines with other groups in order to take the field of human cardiac reprogramming forward.

## Data Availability

The original contributions presented in the study are included in the article/Supplementary Material, further inquiries can be directed to the corresponding author.
