# Supplementary material for: Generation of NKX2.5GFP Reporter Human iPSCs and Differentiation Into Functional Cardiac Fibroblasts
Source: Front Cell Dev Biol. 2022 Jan 21;9:797927. doi: 10.3389/fcell.2021.797927 (PMC8815860; doi:10.3389/fcell.2021.797927)
Supplement: Supplementary file 3 [file DataSheet2.docx]

**Generation of NKX2.5^GFP^ reporter human iPSCs and differentiation into functional cardiac fibroblasts**

**Supplemental data**

**Supplementary Fig. 1: Selection of NKX2.5^GFP^** **human hiPSC clones. a)** Fragment of the Exon 2 sequence of NKX2.5, where the end of the coding sequence (CDS) and STOP codon is shown. The three custom-designed sgRNAs with a PAM sequence (NGG, where N is any nucleotide) close to the STOP codon are represented. **b)** Cleavage efficiency analysis of three different sgRNAs in nucleofected HFF-1 fibroblasts. Dot plot diagram showing the percentage of GFP^+^ cells in non-nucleofected and nucleofected human fibroblasts one day after transduction (left panels); chromatogram obtained by Sanger sequencing of the DNA region close to the expected cleavage site where sgRNA sequence location (black bar) and double peaks corresponding to wild-type and aberrant sequence can be observed (middle panels); and TIDE analysis of DNA sequences obtained from cells nucleofected with each sgRNA where the percentage of cleavage efficiency of each sgRNA relative to the percentage of transduced cells is shown (left panels). **c)** KI at NKX2.5 locus verification in nucleofected hiPSC clones. Seven out of seventy-two clones derived from the pool of nucleofected and Puro resistant hiPSC contained the expected band size after PCR amplification of gDNA with primers directed to sequences located in the endogenous NKX2.5 gene and exogenous donor DNA (represented with arrows in the top scheme). **d)** PCR amplification of gDNA from selected hiPSC clones, with primers directed to sequences of NKX2.5 endogenous gene located at 5´and 3´of the expected donor DNA insertion site (represented with arrows in the top scheme), showed that all CBiPS5 NKX2.5^GFP^ clones presented monoallelic insertion of donor DNA in NKX2.5 gene.

**Supplementary Fig. 2: Analysis of NKX2.5^GFP^ reporter activity. a)** Gene expression analysis of NKX2.5 target genes (RSPO3, EDG7, TGFR3, CEND1) in cardiomyocytes derived from CBiPS5 NKX2.5^GFP^ (white bars) and parental hiPSC line (black bars) at day 20 of cardiac differentiation. **b)** Gene expression of GFP and NKX2.5 at days -4, 5, 7, 9, 11 and 20 of cardiac differentiation in CBiPS5 NKX2.5^GFP^ (white bars) and parental CBiPS5 cell line (black bars). In a-b mean ± SD represented of three technical replicates is represented. **c)** NKX2.5 (in red) and GFP (in green) co-expression analyses by immunocytochemistry in GFP^+^ cells derived from CBiPS5 NKX2.5^GFP^ cells sorted at day 11 of cardiac differentiation (right panels). Undifferentiated CBiPS5 NKX2.5^GFP^ cells were used as negative controls (left panels). Nuclei: DAPI. Scale bars, 100µm. **d)** Gene expression analysis of specific cardiac lineage markers in NKX2.5-GFP^+^ (green circles) and NKX2.5-GFP^-^ cell populations (black circles) sorted at day 11 of cardiac differentiation. **e)** NKX2.5 and GFP gene expression analysis in NKX2.5-GFP^+^ and NKX2.5-GFP^-^ derived cell progeny at day 30 of cardiac differentiation. In d-e, mean of three technical replicates in two biological replicates (Exp1 and Exp2) are represented; the *p* value is annotated in each graph, GFP^-^ group vs GFP^+^ group using Nested-t test. **f)** Gene expression analyses of nodal-like CM markers SHOX2 and TBX3 in NKX2.5-GFP^+^ (green bars) and NKX2.5-GFP^-^ (black bars) derived CM selected with lactate (from Exp1) at day 30 of cardiac differentiation. Mean ± SD represented of three technical replicates is shown.

**Supplementary Fig. 3: KI of GFP at NKX2.5 locus verification in nucleofected BJ-TERT fibroblast clones. a)** Human TERT expression analyses by qRT-PCR in infected BJ fibroblasts. Mean ± SD represented of three technical replicates is represented. **b)** Two clones derived from the pool of nucleofected fibroblasts contained the expected band size (white arrows) after PCR amplification of gDNA with primers directed to sequences located in the endogenous NKX2.5 gene and exogenous donor DNA (represented with arrows in the top scheme). **c)** KI at NKX2.5 locus verification in NKX2.5^GFP^ CBiPS5 derived cFib (NKX2.5-GFP^+^-cFib) analyzed by PCR as described in 3b. White arrows point to the expected PCR band size.

**
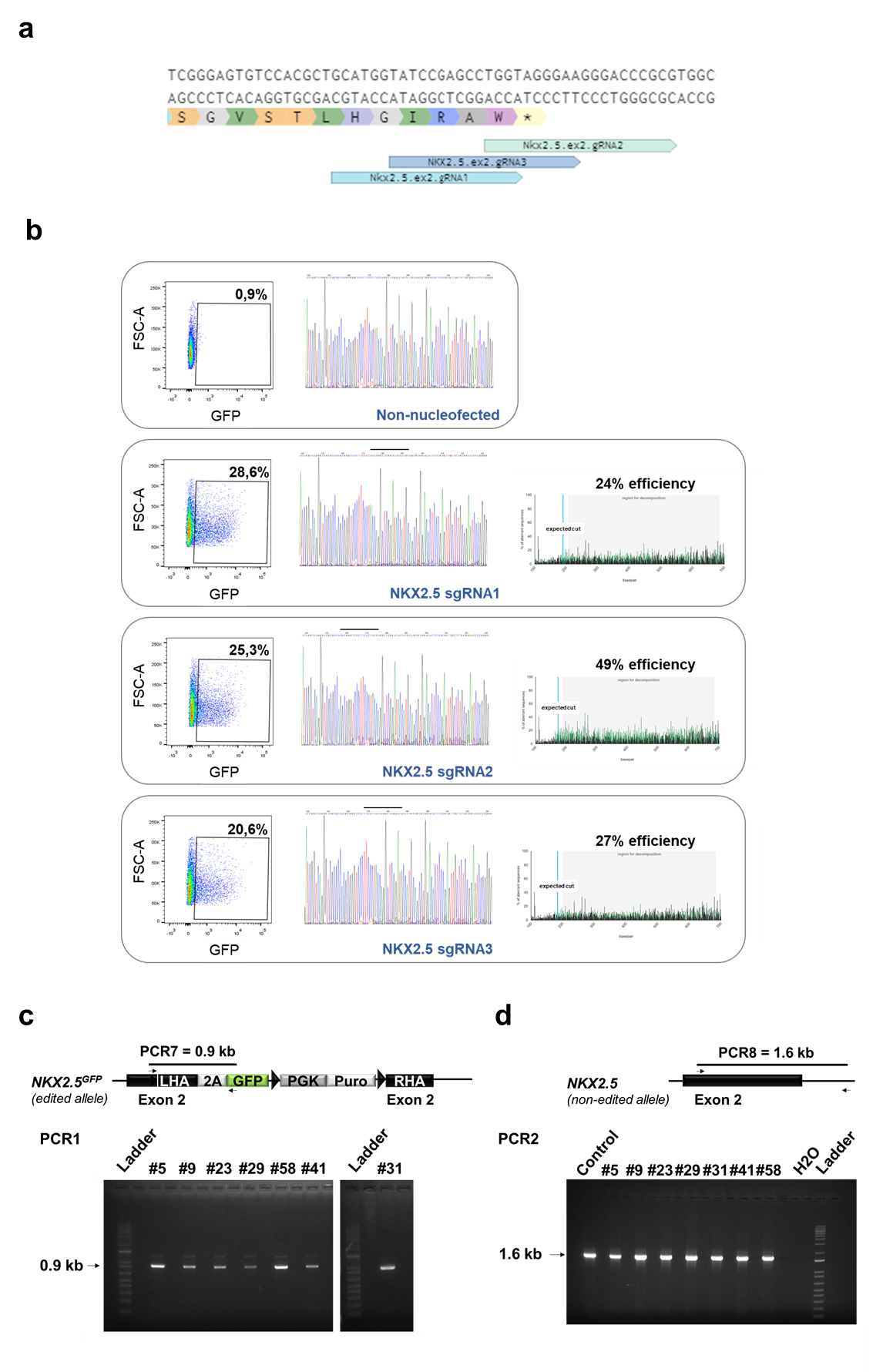
**

**SUPPLEMENTARY FIG. 1**

**
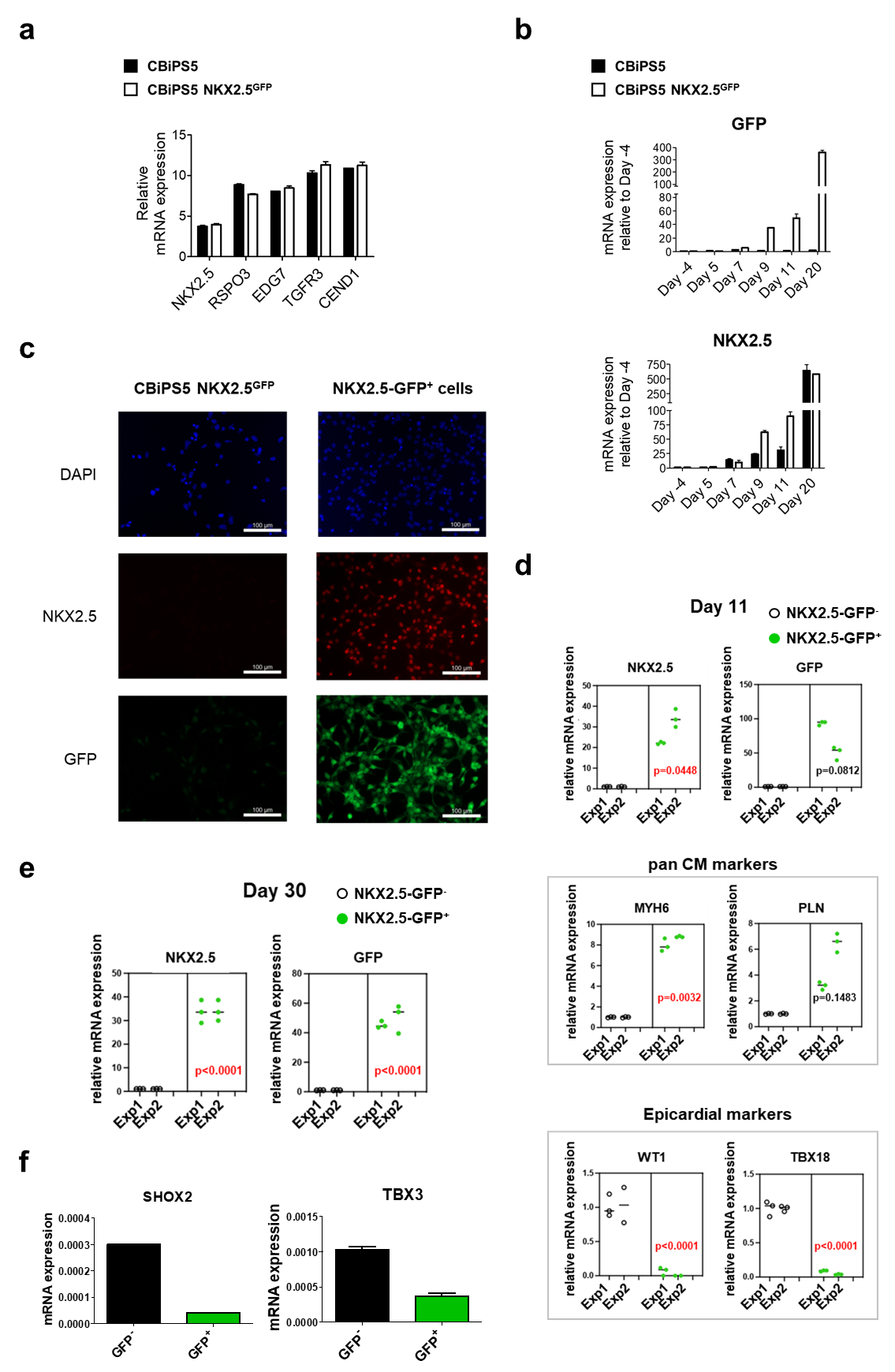
SUPPLEMENTARY FIG. 2**

**
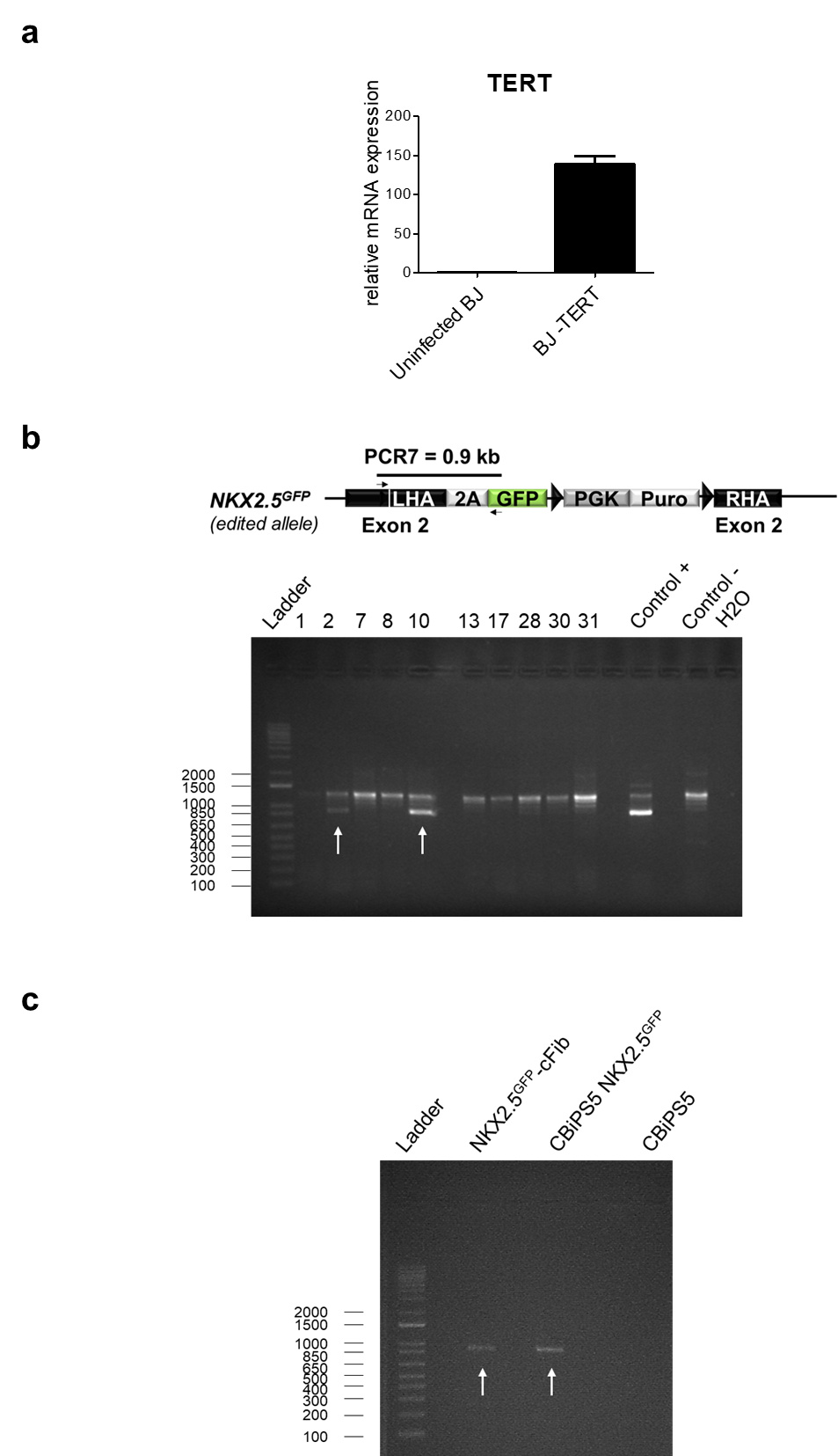
**

**SUPPLEMENTARY FIG. 3**

| **Name** | **Forward (5’- 3’)** | **Reverse (3’- 5’)** | **Product size (bp)** |
| --- | --- | --- | --- |
| NKX2.5-LHA (PCR1) | aatt**gctagc**AGCTGTGCGCGCTGCAGAAG | CCTCTGCCCTCTCCACTGCCCCAGGCTCGGATACCATGCAG | 668 |
| 2A-GFP  (PCR2) | CTGCATGGTATCCGAGCCTGGGGCAGTGGAGAGGGCAGAGG | TCCCCAGCATGCCTGCTATTc | 1039 |
| NKX2.5-LHA-2A-GFP  (PCR3: Fusion PCR) | aatt**gctagc**AGCTGTGCGCGCTGCAGAAG | TCCCCAGCATGCCTGCTATTc | 1666 |
| LoxP-PGK-Puro-LoxP  (PCR4) | aatt**GCGGCCGC**attctagttgtgctcgagagg | aatt**catatg**GGTGCTAGtCCCGGGTCTAGc | 2656 |
| NKX2.5-RHA  (PCR5) | **catatg**ACCGATCCCACCTCAACAGC | **ATGCAT**gaaccagtatggttccagcaa | 602 |
| sgRNA1 | caccGCATGGTATCCGAGCCTGGT | aaacACCAGGCTCGGATACCATGc |  |
| sgRNA2 | caccgTGGTAGGGAAGGGACCCGCG | aaacCGCGGGTCCCTTCCCTACCAc |  |
| sgRNA3 | caccgTATCCGAGCCTGGTAGGGAA | aaacTTCCCTACCAGGCTCGGATAc |  |
| Cleavage site region (PCR6) | aattgctagcAGCTGTGCGCGCTGCAGAAG | CTCATTGCACGCTGCATAAT | 857 |
| SeqTIDE NKX2.5 (Sequencing) |  | CTCATTGCACGCTGCATAAT |  |
| On-target KI  (PCR7) | GTCAAGCCGCTCTTACCAAG | GAACTTCAGGGTCAGCTTGC | 888 |
| On-target KI (Sequencing) | CTCATTGCACGCTGCATAAT |  |  |
| Monoallelic insertion  (PCR8) | GTCAAGCCGCTCTTACCAAG | atctgaggagcctgagaacg | 1691 |

**Supplementary Table 1.** List of primers used for donor DNA generation and knock-in verification. Primers to PCR amplify NKX2.5-LHA (PCR1), 2A-GFP (PCR2), NKX2.5-LHA-2A-GFP (PCR3), LoxP-PGK-Puro-LoxP (PCR4) and NKX2.5-RHA (PCR5) DNA sequences are shown. Restriction enzyme sequences are marked in bold: Nhe I (**gctagc**); Not I (**GCGGCCGC**); NdeI (**catatg**); Nsi I (**ATGCAT**). The primers used for sgRNAs cloning are shown. Primers used to analyze the cleavage efficiency of sgRNAs (PCR6), correct insertion of donor template at NKX2.5 locus (PCR7) and if the insertion was mono- or bi-allelic (PCR8) are shown, including primers used for Sanger sequencing.

| **Component** | **Final concentration** | **Brand** |
| --- | --- | --- |
| **DMEM high glucose (4.5g/l)** | Up to final volume | Sigma-Aldrich |
| **Human Serum Albumin (HSA)** | 500 µg/mL | Millipore |
| **Linoleic acid** | 0,6 µM | Millipore |
| **Lecithin** | 0,6 µM | Millipore |
| **Ascorbic acid** | 50 µg/mL | Millipore |
| **Glutamax** | 7,5 mM | Gibco |
| **Hydrocortisone Hemisuccinate** | 1 µg/mL | Millipore |
| **Rh Insulin** | 5 µg/mL | Millipore |
| **Penicillin/Streptomycin** | 100 U/mL Pen, 100 μg/mL Strep (1% v/v) | Gibco |

**Supplementary Table 2.** Composition of CFBM medium

| **Gene** | **Forward (5’- 3’)** | **Reverse (3’- 5’)** |
| --- | --- | --- |
| CYCLOPHILIN | GAAGAGTGCGATCAAGAACCCATGAC | GTCTCTCCTCCTTCTCCTCCTATCTTTACTT |
| GAPDH | TGCACCACCAACTGCTTAGC | GGCATGGACTGTGGTCATGAG |
| NKX2.5 | CTCCCAACATGACCCTGAGT | CTCATTGCACGCTGCATAAT |
| GFP | AGAACGGCATCAAGGTGAAC | TGCTCAGGTAGTGGTTGTCG |
| POU5F1 (OCT4) | AACCTGGAGTTTGTGCCAGGGTTT | TGAACTTCACCTTCCCTCCAACCA |
| NANOG | CCTGAAGACGTGTGAAGATGAG | GCTGATTAGGCTCCAACCATAC |
| MYH6 (αMHC) | CTGAAACCGAGAATGGGAAG | CGCTCCTTGAGGTTGAAAAG |
| MYL2 (MLC2v) | CAACGTGTTCTCCATGTTCG | GTCAATGAAGCCATCCCTGT |
| PLN | CCCAGCTAAACACCCGTAAG | AGCTGGCAGCCAAATATGAG |
| IRX4 | AGGGCTATGGCAACTACGTG | CGAACCATCCTTGGAATCAA |
| KCNA5 | CCAGCAGAGGGATAACCCAAAC | TTGGAACACATGGATGGAGGAG |
| NR2F2 | GCAAGTGGAGAAGCTCAAGG | TCCACATGGGCTACATCAGA |
| MYL7 | AACGTGGTTCTTCCAACGTC | AGGTCTGCCTTGCAGATGAT |
| NPPA | GCTGCAGCTTCCTGTCAACACT | AGGCGAGGAAGTCACCATCAA |
| TBX18 | GAAGCGCTTTCATGAGATAGG | GATCTTCACTCTCATTGCTGGA |
| WT1 | GCTCTGAGGATTGTGCAGTG | AATGAGTGGTTGGGGAACTG |
| ACTA2 (αSMA) | GTGTTGCCCCTGAAGAGCAT | GCTGGGACATTGAAAGTCTCA |
| CNN1 | CTGTCAGCCGAGGTTAAGAAC | GAGGCCGTCCATGAAGTTGTT |
| SM22α | AAGAATGATGGGCACTACCG | AAGGCCAATGACATGCTTTC |
| PECAM1 | TGTGCCTGCAGTCTTCACTC | TCAGGTTCTTCCCATTTTGC |
| KDR | AGCGATGGCCTCTTCTGTAA | ACACGACTCCATGTTGGTCA |
| TCF21 | GAAACCCGAGAGTGACCTGA | GCTCCAGGTACCAAACTCCA |
| VIM | TCAGAGAGAGGAAGCCGAAA | ATTCCACTTTGCGTTCAAGG |
| POSTN | GCAACGGAGAGACTCAAGATG | TCTGTTGAAGGGCACAGACA |
| THY1 (CD90) | ACCTACACGTGTGCACTCCA | GCCCTCACACTTGACCAGTT |
| TERT | CGAGCTGCTCAGGTCTTTCT | GCACCCTCTTCAAGTGCTGT |

**Supplementary Table 3.** List of primers used for qRT-PCR analyses using QuantStudio 3 or 5 Real Time PCR Systems (Thermo Fisher Scientific) with PowerUp SYBR Green Master Mix.

| **COL1A1**  Ref: Hs.PT.58.15517795  Primer 1: 5´-TTCTGTACGCAGGTGATTGG-3´  Primer 2: 5´-GACATGTTCAGCTTTGTGGAC-3´  Probe: 5´-/56-FAM/TCGAGGGCC/ZEN/AAGACGAAGACATC/3IABkFQ/-3´ |
| --- |
| **LOX**  Ref: Hs.PT.58.40011520  Primer 1: 5´-AGTGGCTAAACTCATCCATACTG-3´  Primer 2: 5´-GCTCAGATTTCCCAAAGAGT-3´  Probe: 5´-/56-FAM/TGACAACTG/ZEN/TGCCATTCCCAGGA/3IABkFQ/-3´ |
| **FN1**  Ref:  Hs.PT.58.21141138  Primer 1: 5´-GTCCTTGTGTCCTGATCGTTG-3´  Primer 2: 5´-AGGCTGGATGATGATGGTAGATTG-3´  Probe: 5´-/56-FAM/CGTCCGCTG/ZEN/CCTTCTCCCA/3IABkFQ/-3´ |
| **RNA18S5**  Ref:  Hs.PT.39a.22214856.g  Primer 1: 5´-GGACATCTAAGGGCATCACAG-3´  Primer 2: 5´-GAGACTCTGGCATGCTAACTAG-3´  Probe: 5´-/56-FAM/TGCTCAATC/ZEN/TCGGGTGGCTGAA/3IABkFQ/-3´ |

**Supplementary Table 4.** List of primers used for qRT-PCR analyses using AriaMx Real-Time PCR System (Agilent Technologies) with TaqMan Fast Advanced Master Mix (Applied Biosystems).

| **Antibody** | **Type, clonality** | **Host** | **Usage** | **Brand** | **Reference** |
| --- | --- | --- | --- | --- | --- |
| **NKX2.5** | Primary, Polyclonal | Goat | 1:500 | Santa Cruz | sc-8697 |
| **GFP** | Primary, Polyclonal | Chicken | 1:500 | Aves Labs | GFP-1020 |
| **α-SMA** | Primary, Monoclonal | Mouse | 1:100 | Sigma-Aldrich | A5228 |
| **Fibronectin** | Primary, Monoclonal | Mouse | 1:400 | Sigma-Aldrich | F6140 |
| **Collagen I** | Primary, Polyclonal | Rabbit | 1:10 | BioRad | 2150 0020 |
| **Alexa Fluor 555, anti-rabbit IgG** | Secondary, Polyclonal | Goat | 1:500 | ThermoFisher scientific | A21428 |
| **Alexa Fluor 555, anti-mouse IgG** | Secondary, Polyclonal | Goat | 1:500 | ThermoFisher scientific | A32727 |
| **Alexa Fluor 594, anti-goat IgG** | Secondary, Polyclonal | Donkey | 1:500 | ThermoFisher scientific | A11058 |
| **Alexa Fluor 488, anti-chicken IgG** | Secondary, Polyclonal | Goat | 1:500 | ThermoFisher scientific | A11039 |

**Supplementary Table 5.** Description of the antibodies used for immunostaining.
